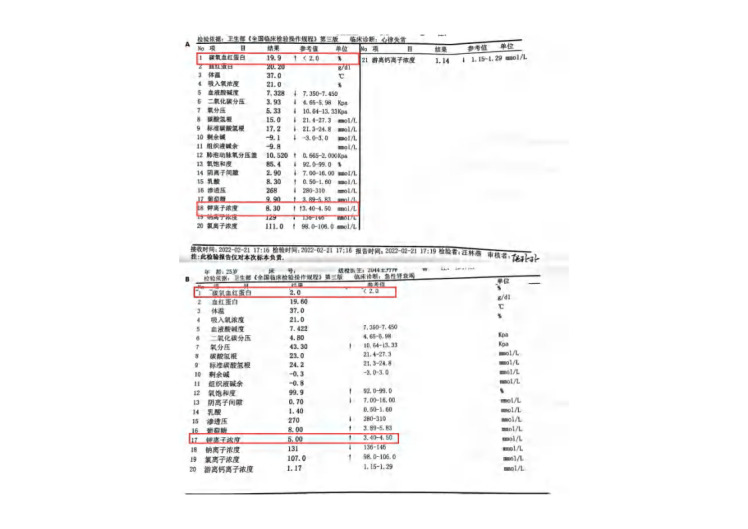# Hyperkalemia combined with carbon monoxide poisoning, should hemodialysis or hyperbaric oxygen treatment be given first?: A CARE-compliant article

**DOI:** 10.1097/MD.0000000000042934

**Published:** 2025-06-27

**Authors:** Lingling Tuo, Aifeng Zhang, Xinyun Li, Jiejin Zhao

**Affiliations:** a The First People’s Hospital of Xiaoshan District, Hangzhou, China; b School of Rehabilitation, Hangzhou Medical College, Hangzhou, China.

**Keywords:** carbon monoxide poisoning, hemodialysis, hyperbaric oxygen, hyperkalemia, sequential therapy

## Abstract

**Rationale:**

Hyperkalemia and acute carbon monoxide (CO) poisoning are both life-threatening conditions. When these conditions coexist, there is a debate on whether hemodialysis or hyperbaric oxygen (HBO) therapy should be prioritized. Our discussion suggests that HBO therapy may help by lowering residual carbon dioxide levels and correcting electrolyte imbalances, consequently decreasing the risks linked to both CO poisoning and hyperkalemia.

**Patient concerns:**

A 25-year-old man presented with hyperkalemia and CO poisoning. He was promptly taken to the emergency room where he received immediate interventions including intravenous infusion of 5% sodium bicarbonate solution, glucose plus insulin, diuretics, and calcium supplementation in a HBO environment. Subsequently, hemodialysis was performed. Following this treatment protocol, the patient showed significant improvements in hyperkalemia, CO levels, general weakness, cardiac arrhythmias, and other symptoms. This sequential therapy effectively eliminated the remaining CO in his system and addressed the electrolyte imbalances crucial to his recovery. After 4 weeks of comprehensive treatment, the patient’s hyperkalemia and CO poisoning issues were successfully resolved.

**Diagnoses:**

Based on the thorough history, electrocardiographs and the laboratory tests.

**Interventions:**

The interventions included intravenous infusion of 5% sodium bicarbonate solution, glucose plus insulin, diuretics, calcium supplementation in a HBO environment, and followed by hemodialysis.

**Outcomes:**

The following clinical improvements were detected: consciousness was clear; the cardiac arrhythmia symptoms were improved (electrocardiograph became normal); normal electrolytes with no other abnormalities; displayed no nervous system symptoms.

**Lessons:**

Sequential treatment of intravenous fluid rehydration, HBO, hemodialysis may be effective in treating patients with hyperkalemia combined with CO poisoning.

## 1. Introduction

Acute hyperkalemia and carbon monoxide (CO) poisoning are both fatal conditions. Hyperkalemia can lead to cardiac arrest, limb and perioral sensations, numbness, extreme fatigue, muscle aches, and more. On the other hand, CO poisoning is a common and potentially lethal condition that can result in hypoxic injury and delayed neurological sequelae. Hemodialysis is frequently used clinically to address acute hyperkalemia, while hyperbaric oxygen therapy (HBOT) is commonly employed in treating CO poisoning. Both treatments typically require more than 1 hour of treatment time. Therefore, the decision-making process regarding the choice of method and sequence of treatments represents a critical area of medical research.

The interaction between CO poisoning and hyperkalemia is not fully understood, but the stress and cell damage from CO may lead to potassium release, potentially worsening the patient’s condition. Research is ongoing to investigate how HBOT impacts potassium levels, with suggestions that the treatment could stabilize cell membranes and decrease potassium release. Limited studies exist on the effects of hemodialysis on CO poisoning. This presents a challenge for clinicians in deciding the optimal treatment sequence for patients with hyperkalemia and CO poisoning.

When studying the combination of hyperkalemia and CO poisoning, the order of performing HBOT or hemodialysis is a critical consideration. This study opted for intravenous infusion of 5% sodium bicarbonate solution, glucose plus insulin, diuretics, and calcium supplementation in a HBO setting, followed by hemodialysis. The results showed significant efficacy. The increased atmospheric pressure and oxygen levels may help counteract the effects of CO on the brain and heart, lowering the risk of delayed neurological complications, and managing hyperkalemia-related risks. This study is vital for refining treatment strategies to enhance outcomes for patients with this complex and dangerous condition.

## 2. Case report

On February 21, 2022, a 25-year-old Chinese male attempted suicide by inhaling CO for 3 hours and ingesting 30 tablets containing potassium chloride. He was found unconscious by his family 2 hours later and was promptly rushed to the First People’s Hospital of Xiaoshan District.

**Figure 1.**
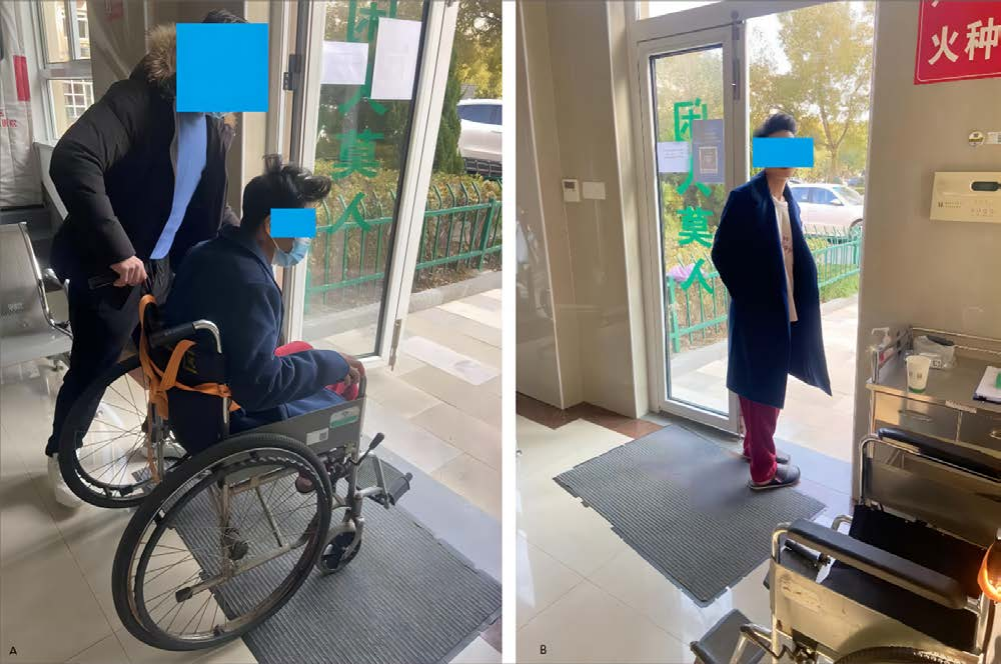
The patients mental state before and after treatment. (A) Before the treatment, the patient was weakness and unable to move independently; (B) after the treatment, the patient walk normally.

**Figure 2.**
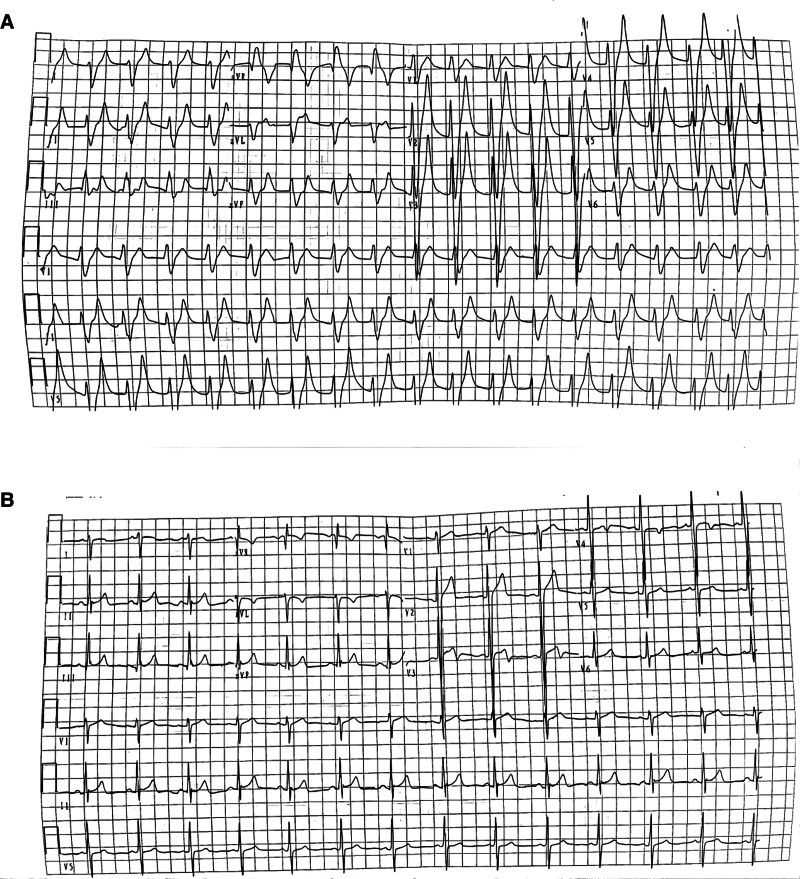
The ECG of the patient before and after the treatment. (A) Before the treatment; (B) after the treatment. ECG = electrocardiogram.

Following admission, thorough medical evaluation, and emergency intervention, the patient presented with severe physical weakness, palpitations, and an inability to move independently, as illustrated in Figure 1A. The electrocardiogram (ECG) findings, depicted in Figure 2A, indicated various cardiac issues including atrioventricular or branch blocks, nonspecific intraventricular conduction delay, alterations in T waves, prolonged PR interval, shortened QT interval, and reduced P wave amplitude. Blood gas analysis showed a carboxyhemoglobin level of 19.9% and a potassium ion concentration of 8.30 mmol/L, as shown in Table S1A, Supplemental Digital Content, https://links.lww.com/MD/P272. Additionally, the serum brain natriuretic peptide concentration was measured at 2310.5 ng/L, and fast troponin fibers type T level was 0.087 µg/L, as demonstrated, respectively. His head CT scan appeared normal.

In response to these findings, the medical team promptly initiated intravenous infusion of 5% sodium bicarbonate solution, glucose plus insulin, diuretics, and calcium supplementation in a HBO setting, followed by hemodialysis to address hyperkalemia and CO poisoning, and also mitigate associated health risks. Due to the gravity of his condition upon admission, the sequence of interventions played a crucial role in his treatment.

**Figure 3.**
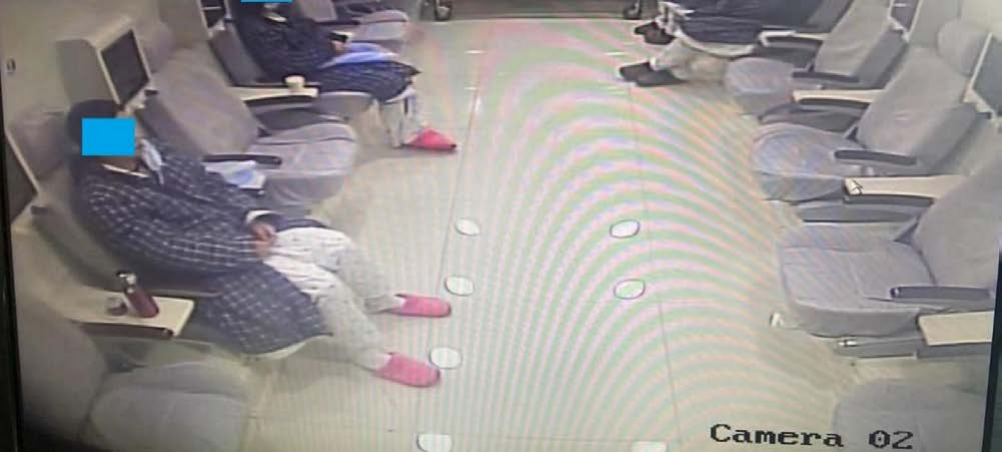
Patients are accepting the hyperbaric oxygen treatment.

Firstly, the intravenous infusion of 5% sodium bicarbonate solution, glucose plus insulin, diuretics, and calcium supplementation in a HBO setting. The HBO chamber was set to a gauge pressure of 0.1 MPa, equating to an effective pressure of 2 atmospheres. Each session lasted 100 minutes, incorporating 40 minutes for gradual pressure modulation and 60 minutes for the oxygen treatment itself. The regimen consisted of daily treatments, 5 days a week. Figure 3 illustrates patients receiving HBOT during their scheduled sessions.

Following HBOT, a temporary central venous catheter was inserted using the wire-guided insertion method, known as the Seldinger technique. The procedure involved positioning the patient in a supine position with the head down (Trendelenburg position), disinfecting the skin at the puncture site, laying a sterile towel, and donning sterile gloves. Local infiltration anesthesia with 0.5% lidocaine was administered, followed by venipuncture using a trocar needle. Once the internal jugular vein was accessed, venous blood was drawn. The trocar needle was then removed, leaving the cannula in place. A guide wire was inserted through the cannula, and after removing the trocar, a dilator was used to expand the tissues, including the skin, subcutaneous tissue, and central veins, along the guide wire. The appropriate catheter was selected, each cavity was filled with heparin saline, and it was inserted into the central vein along the guide wire. The guide wire was then removed, and the patency of each catheter cavity was verified. Finally, each cavity was filled with 0.2 mg/mL heparin saline, capped with a heparin cap, the catheter was secured to the skin with sutures, and local sterile dressing was applied.

During hemodialysis treatment lasting 4 hours, unfractionated heparin was used as an anticoagulant. The initial dose administered was 0.5 mg/kg, followed by additional doses of 10 mg/h through continuous intravenous infusion. Heparin administration was stopped 30 minutes before the end of hemodialysis. A blood flow rate >250 mL/min was maintained, and the post-dilution replacement method was employed.

Then, the patient exhibited clear cognitive function and was capable of independent activity (as seen in Fig. 1B). The ECG presented a normal sinus rhythm (Fig. 2B). Laboratory tests revealed a carboxyhemoglobin level of 2.0%, a potassium ion concentration of 5.0 nmol/L (Table S1B, Supplemental Digital Content, https://links.lww.com/MD/P272), the serum brain natriuretic peptide concentration of 777.3 ng/L, and a troponin T level of 0.053 µg/L.

To reduce the risk of delayed encephalopathy after carbon monoxide poisoning (DEACMP), a series of 20 HBO sessions were prescribed. Following the complete course, the patient reported no discomfort and displayed no symptoms related to cardiac or neurological conditions.

## 3. Discussion

Hyperkalemia, characterized by elevated levels of potassium ions in the blood is linked to an increased risk of serious health events, such as ventricular arrhythmias and sudden cardiac death.^[1]^ This condition significantly affects neuromuscular and cardiac functions, leading to a wide range of symptoms including cardiovascular and neuromuscular issues, abnormal ECG readings, and disturbances in consciousness.

Additionally, CO poisoning is a common cause of accidental harm, notably prevalent in northern China.^[2,3]^ Typically, 2 to 60 days post-exposure, individuals recovering from acute CO poisoning begin to show symptoms of what is known as DEACMP. This condition manifests in various neurological impairments, including cognitive disturbances, damage to the motor system, and cortical dysfunction.^[4]^

Currently, treatments for CO poisoning include HBOT and other methods like pulmonary phototherapy and veno-venous extracorporeal blood phototherapy.^[5,6]^ DEACMP is typically treated with medications such as dexamethasone and acetylcholinesterase inhibitors. For managing hyperkalemia, clinical guidelines recommend interventions like intravenous insulin–dextrose, potassium binders, and inhibitors of the renin–angiotensin–aldosterone system.^[7–9]^

Despite these treatments, there is a notable lack of robust evidence supporting effective interventions for patients suffering from both CO poisoning and hyperkalemia, especially in emergency situations. This report highlights a case in which a patient suffering from both conditions was effectively treated with a combination of HBOT and hemodialysis.

For the patients who are in need of emergency treatment, choosing the right treatment sequence as critical. Hyperkalaemia is a potential life-threatening electrolyte abnormality. The impact of plasma potassium normalization is the critical factor. In patients with cardiac disease or abnormal cardiac function, hyperkalaemia determined at admission have a negative prognostic impact on survival.^[4]^ This means that the heart muscle is damaged. However, nowadays the hemodynamic-targeted treatment brings some advantages, such as dialysis dependence, junctional rhythm, concomitant presentation with hypothermia, acidemia, or sepsis.^[10]^

CO poisoning and hyperkalemia are lethal disease. The mortality is very high.^[11,12]^ CO poisoning will not just cause brain damage and DEACMP, but also lead to a midwall injury of the heart, which is related to acute myocardial dysfunction and subacute deterioration of myocardial strain.^[13]^ Treatment options for CO poisoning remain controversial. Some data suggests that in acute severe CO poisoning, patients who were treated using therapeutic hypothermia combined with HBOT had significantly more favorable neurocognitive outcomes than those treated with HBOT alone.^[14]^ At the same time, there are still a certain number of doctors recommend HBO for CO poisoning.^[15]^

In this case, we thought that HBOT appears to improve both neural and cardiac performance.^[16]^ While normobaric oxygen lacks in efficacy in decreasing the concentration of carboxyhemoglobin in the body, the HBO is effective. Study for eliminating the CO by treating blood extracorporeally at elevated oxygen partial pressure, and finds that the CO was declined.^[17]^ With the further researches of HBO, mechanisms are explored, such as inhibiting apoptosis and inflammation, antioxidative, promoting angiogenesis.^[18–20]^ HBOT has shown promising results in reducing CO levels by enhancing the oxygen-carrying capacity of the blood, which helps to displace CO from hemoglobin. This method is particularly effective in emergency settings where rapid detoxification is crucial. The decline in CO levels observed in these studies underscores the potential of HBOT as an effective treatment modality.

Therefore, when we faced the patient with hyperkalemia and CO poisoning, we first choose intravenous treatment with HBO, and then perform hemodialysis.

Based on the above case, we speculate that HBO is the first choice followed by hemodialysis for successful treatment. The possible mechanisms are as follows.

A notable attempt was made in this study to administer intravenous infusion of 5% sodium bicarbonate solution, glucose plus insulin, diuretics, and calcium supplementation in conjunction with HBOT. This combined approach is likely to enhance the efficacy of these intravenous drugs, leading to positive outcomes such as mitigating the toxic effects of potassium on the heart, preserving heart function, and facilitating potassium excretion. In addition to preparing for hemodialysis treatment, it is important to protect kidney function, increase glomerular filtration rate, improve mineral metabolism disorders, control blood pressure, and correct abnormalities in lipid metabolism, glucose metabolism, and hyperuricemia.

HBOT may indirectly affect the levels of potassium ions in the body through 2 main mechanisms: affecting the permeability of cell membranes and activating specific metabolic pathways.

### 3.1. Restore the function of ion pump in cell membrane

Under hypoxia, the activity of Na⁺/K⁺-ATP enzyme in cell membrane is impaired, leading to the outflow of K⁺ in cells and the increase of serum K⁺.^[21]^ HBOT improves tissue oxygen supply, restores ATP synthesis, enhances the function of ion pump, and promotes the active transport of K⁺ into cells, thus reducing blood potassium concentration.^[22]^

### 3.2. Inhibition of acidosis-mediated K⁺ release

CO poisoning and hypoxia are often accompanied by lactic acidosis, leading to changes in cell membrane permeability and K⁺ outflow.^[23]^ HBOT can correct hypoxia, reduce lactic acid produced by anaerobic metabolism, stabilize intracellular pH value, and inhibit the release of K⁺ related to acidosis.

### 3.3. Regulates the adrenergic system

Hyperbaric oxygen can inhibit the hypoxia-induced excessive secretion of adrenaline, reduce the β receptor mediated intracellular K⁺ excretion, and reduce the sympathetic nerve excitability to potassium distribution.^[24]^

Although these mechanisms provide a theoretical explanation, the actual impact of HBOT on potassium levels remains complex and is influenced by many factors, including the duration of treatment, pressure levels, the patient’s initial health condition, and their specific medical background. Furthermore, the evidence for these potential effects mainly comes from animal studies and limited clinical observations, and more research is needed to clarify how HBO accurately regulates potassium levels in the human body. Therefore, when considering using HBOT as a treatment method to affect potassium levels, caution should be exercised and should be conducted under the supervision of professional medical personnel.

This case report has a limitation in that, while we proposed a feasible treatment sequence, we were unable to determine the efficacy of HBOT for hyperkalemia combined with CO poisoning. Therefore, higher-quality studies are needed to confirm the effectiveness, safety and mechanism of this therapy, especially when multidisciplinary teams (including emergency physicians, nephrologists, cardiologists, poisoning specialists, etc) important role in the case.

## Author contributions

**Conceptualization:** Jiejin Zhao.

**Data curation:** Lingling Tuo, Jiejin Zhao.

**Funding acquisition:** Xinyun Li.

**Investigation:** Aifeng Zhang.

**Methodology:** Aifeng Zhang.

**Project administration:** Xinyun Li, Jiejin Zhao.

**Resources:** Aifeng Zhang.

**Writing – original draft:** Lingling Tuo.

**Writing – review & editing:** Lingling Tuo, Aifeng Zhang.

## Supplementary Material

**Figure:**